# *p*-Cymene and Rosmarinic Acid Ameliorate TNBS-Induced Intestinal Inflammation Upkeeping ZO-1 and MUC-2: Role of Antioxidant System and Immunomodulation

**DOI:** 10.3390/ijms21165870

**Published:** 2020-08-15

**Authors:** Rodrigo de Oliveira Formiga, Edvaldo Balbino Alves Júnior, Roseane Carvalho Vasconcelos, Gerlane Coelho Bernardo Guerra, Aurigena Antunes de Araújo, Thaís Gomes de Carvalho, Vinícius Barreto Garcia, Raimundo Fernandes de Araújo Junior, Francisco Allysson Assis Ferreira Gadelha, Giciane Carvalho Vieira, Marianna Vieira Sobral, José Maria Barbosa Filho, Fernando Spiller, Leônia Maria Batista

**Affiliations:** 1 Postgraduate Program in Natural and Synthetic Bioactive Products, Health Sciences Center, Federal University of Paraíba (UFPB), João Pessoa 58051970, Brazil; rodrigo.formigadc@gmail.com (R.d.O.F); edvaldojunioralves@gmail.com (E.B.A.J.); allyssonassis@hotmail.com (F.A.A.F.G.); giciane.carvalho@uol.com.br (G.C.V.); mariannavbs@gmail.com (M.V.S.); jbarbosa@ltf.ufpb.br (J.M.B.F.); 2 Department of Biophysics and Pharmacology, Biosciences Center, Federal University of Rio Grande do Norte, Natal 59064-741, Brazil; r.roseane@hotmail.com (R.C.V); gerlaneguerra@hotmail.com (G.C.B.G.); aurigena@ufrnet.br (A.A.d.A.); 3 Department of Morphology, Histology and Basic Pathology, Biosciences Center, Federal University of Rio Grande do Norte, Natal 59064-741, Brazil; thaisbida2011@hotmail.com (T.G.d.C.); vbgbiomed@gmail.com (V.B.G.); araujojr@cb.ufrn.br (R.F.d.A.J.); 4 Department of Pharmacology, Federal University of Santa Catarina (UFSC), Florianópolis 88037-000, Brazil; spiller.farmaco@gmail.com

**Keywords:** *p*-cymene, rosmarinic acid, intestinal inflammation, antioxidant, immunomodulation, cytoprotection

## Abstract

*p*-Cymene (*p*-C) and rosmarinic acid (RA) are secondary metabolites that are present in medicinal herbs and Mediterranean spices that have promising anti-inflammatory properties. This study aimed to evaluate their intestinal anti-inflammatory activity in the trinitrobenzene sulphonic acid (TNBS)-induced colitis model in rats. *p*-C and RA (25–200 mg/kg) oral administration reduced the macroscopic lesion score, ulcerative area, intestinal weight/length ratio, and diarrheal index in TNBS-treated animals. Both compounds (200 mg/kg) decreased malondialdehyde (MDA) and myeloperoxidase (MPO), restored glutathione (GSH) levels, and enhanced fluorescence intensity of superoxide dismutase (SOD). They also decreased interleukin (IL)-1β and tumor necrosis factor (TNF)-α, and maintained IL-10 basal levels. Furthermore, they modulated T cell populations (cluster of differentiation (CD)4^+^, CD8^+^, or CD3^+^CD4^+^CD25^+^) analyzed from the spleen, mesenteric lymph nodes, and colon samples, and also decreased cyclooxigenase 2 (COX-2), interferon (IFN)-γ, inducible nitric oxide synthase (iNOS), and nuclear transcription factor kappa B subunit p65 (NFκB-p65) mRNA transcription, but only *p*-C interfered in the suppressor of cytokine signaling 3 (SOCS3) expression in inflamed colons. An increase in gene expression and positive cells immunostained for mucin type 2 (MUC-2) and zonula occludens 1 (ZO-1) was observed. Altogether, these results indicate intestinal anti-inflammatory activity of *p*-C and RA involving the cytoprotection of the intestinal barrier, maintaining the mucus layer, and preserving communicating junctions, as well as through modulation of the antioxidant and immunomodulatory systems.

## 1. Introduction

Inflammatory bowel diseases (IBD), such as ulcerative colitis (UC) and Crohn’s disease (CD), are a group of recurrent and debilitating disorders [1,2]. They present a high prevalence in Western countries in up to 0.5% in the general population [3], affecting around 6.8 million each year [4]. The incidence of IBD is growing mostly in developing countries, particularly in Brazil, where the annual prevalence has increased by over 6.4% [5]. In this sense, they may represent a relevant social and economic burden on governments and health services in the upcoming years [4].

Although the cause of IBD remains unknown, the observed intestinal disorders seem to be associated with immune impairment, unbalanced microbiota, exposure to environmental factors, and genetic susceptibility [6]. In particular, UC displays colon-restricted lesions that appear continuously on mucosa and submucosa layers characterized by a Th2-like and natural killer T cell (NKT) phenotype [7,8,9,10], accompanied by neutrophil infiltration into the lamina propria; edema; increased T cell reactivity; and depletion of mucins, such as mucin-type 2 (MUC-2) [11,12,13]. This results in instabilities of the intestinal barrier structure formed by epithelial cells that are connected by tight junction structural proteins (claudin, occludin, zonula occludes, etc.) [14]. In this context, the imbalance between pro-inflammatory (tumor necrosis factor (TNF), interferon (IFN)-γ, interleukin (IL)-6, IL-12, IL-21, IL-23, IL-17), and anti-inflammatory (IL-10, TGF-β, IL-35) cytokines in the intestinal microenvironment is the hallmark of IBD and plays a key role in tissue damage and chronic inflammation [15]. The release of cytokines contributes to the overproduction of reactive oxygen species (ROS) and the impairment of endogenous antioxidant mechanisms promoted by glutathione (GSH), superoxide dismutase (SOD), and other molecules, being tightly involved with the development of tissue injury [16]. These miscellaneous pathological changes result in common clinical manifestations including anal lesions, rectal bleeding, diarrhea, lower abdominal pain, and tenesmus, which dramatically impact quality of life [17].

Therapy of IBD is centered on aminosalicylates, immunosuppressants, and immunobiologicals, and sometimes surgical interventions [18,19], which result in large costs for health services [20]. Moreover, drug therapy is constantly associated with several side effects that contribute to treatment non-adherence [18,21]. Additionally, they constantly fail to prevent IBD active phase relapse [2,22]. To promote new pharmacological approaches, researchers are considering natural products as promising in drug discovery. Indeed, among the 74 novel substances approved by the FDA from 1981 to 2014 with anti-inflammatory, immunomodulatory, and immunosuppressive properties, 33 are classified as macromolecules, unchanged natural products, or their derivatives [23]. In this context, the pharmacological effects of secondary metabolites, such as monoterpenes [24,25] and polyphenols [26], have been demonstrated for IBDs. *p*-Cymene (*p*-C) is a monoterpene present in more than 100 different plant species, and is a component of fruits, wines, and spices, such as *Origanum vulgare* (oregano) and *Thymus vulgaris* (thyme) [27,28], which has previously presented analgesic, anti-inflammatory [29], and antioxidant activities [30]. Rosmarinic acid (RA) is a polyphenol ester of caffeic acid and 3,4-dihydroxyphenyllactic acid that is commonly found in *Melissa officinalis* (lemon balm), *Mentha piperita* (mint), *Salvia officinalis* (sage), and *Rosmarinus officinalis* (rosemary) [31,32]. Pharmacological studies indicate potent antioxidant [33], cytoprotective [34], and anti-inflammatory activities [35].

Considering the pharmacological potential of *p*-C and RA and the lack of studies evaluating the effects of these isolated compounds in IBD models, we aimed to assess their effects on the outcome of trinitrobenzene sulfonic acid (TNBS)-induced ulcerative colitis. Our main findings showed that both drugs ameliorate disease severity. This protection is provided due to immunomodulatory effects, from decreasing cell infiltrate, release of cytokines, modulation of T cells, and also antioxidant activity, which is reflected in the maintenance and integrity of the intestinal barrier.

## 2. Results

### 2.1. p-C and RA Decreased Intestinal Inflammation in a TNBS-Induced Colitis Model

**Figure 1:**
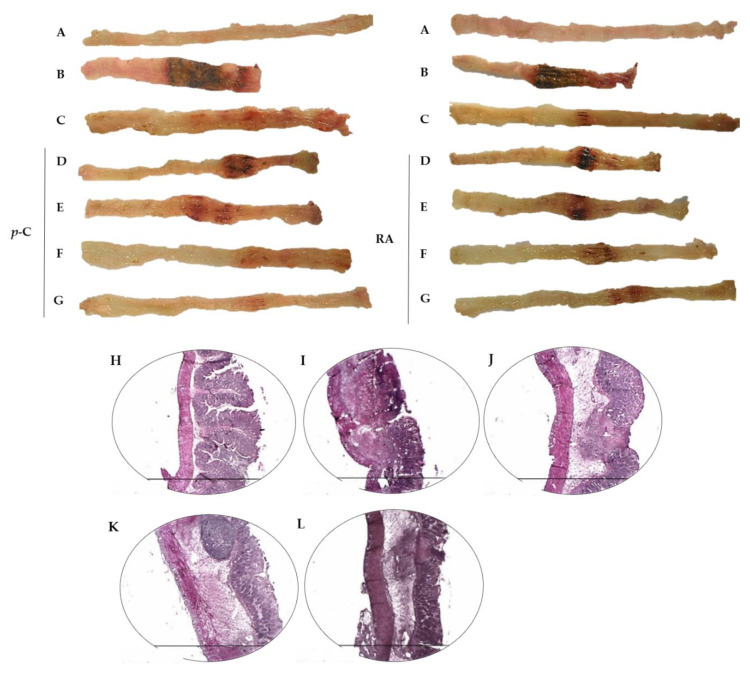
Representative images of rat colons from non-colitic group (**A**); colitic groups (5% Tween 80 or 0.9% NaCl treated groups) (**B**); prednisolone 2 mg/kg (**C**); *p*-C or RA (RA) 25 mg/kg (**D**), 50 mg/kg (**E**), 100 mg/kg (**F**), and 200 mg/kg (**G**) subjected to TNBS-induced ulcerative colitis model. The figure also shows representative hematoxylin–eosin (HE)-stained photomicrographs (*n* = 3 per group) from (**H**) non-colitic; (**I**) colitic; (**J**) prednisolone 2 mg/kg; (**K**) *p*-C 200 mg/kg; and (**L**) RA 200 mg/kg. 40× magnification. Scale: 1500 µm.

The effect of *p*-C and RA was evaluated with a focus on their intestinal anti-inflammatory effect on TNBS-induced inflammation. To do so, rats were pre- (48, 24, and 1 h before TNBS) and post-treated (24 h after TNBS) orally with the vehicles 5% Tween 80 or 0.9% NaCl (colitic groups), prednisolone (2 mg/kg, used as a reference of anti-inflammatory drug), *p*-C (25–200 mg/kg) dissolved in 5% Tween 80, or RA (25–200 mg/kg) dissolved in 0.9% NaCl. The parameters ulcerative area (UA), lesion score, weight/length ratio, and diarrhea were evaluated 48 h after TNBS administration, as shown in Table 1. TNBS administration to colitic groups led to the development of large lesions and intense signs of inflammation with gross ulcerative areas, typically affecting 4 cm of the tissue. These effects were followed by an increased weight/length ratio of the colonic segment and the diarrhea score, which was determined by the evacuation index (EI), when compared to non-colitic group (*p* < 0.001). The treatments with *p*-C (*p* < 0.01; *p* < 0.001), RA (*p* < 0.01; *p* < 0.001), or prednisolone (*p* < 0.001) reduced those parameters in all evaluated doses, showing evident signs of disease recovery (representative images in Figure 1).

The most effective doses of *p*-C and RA (200 mg/kg) were selected for the following experiments. The microscopic evaluation showed that the non-colitic group presented normal characteristic tissue, and well-preserved mucosal muscle, submucosal layer, and external muscle (Figure 1H). However, in the colitic groups, we observed extensive inflammation and ulcerative areas in the mucosal epithelium, mucosal muscle thickness, and the presence of intense inflammatory infiltrate in submucosa with the involvement of external muscle. Submucosa also presented fibrous tissue with leukocyte infiltration (Figure 1I). The groups treated with prednisolone (Figure 1J), *p*-C (Figure 1K), or RA (Figure 1L), the latter to a lesser extent, showed decreased ulcerated mucosal injury, epithelial reconstitution with preservation of intestinal crypts, decreased inflammatory exudate, thinner submucosa layer, and reduced inflammatory infiltrate.

### 2.2. p-C and RA Increased Antioxidant Molecules (GSH and SOD) and Decreased Malondialdehyde (MDA) and Myeloperoxidase (MPO) Levels in a TNBS-Induced Colitis Model

**Figure 2:**
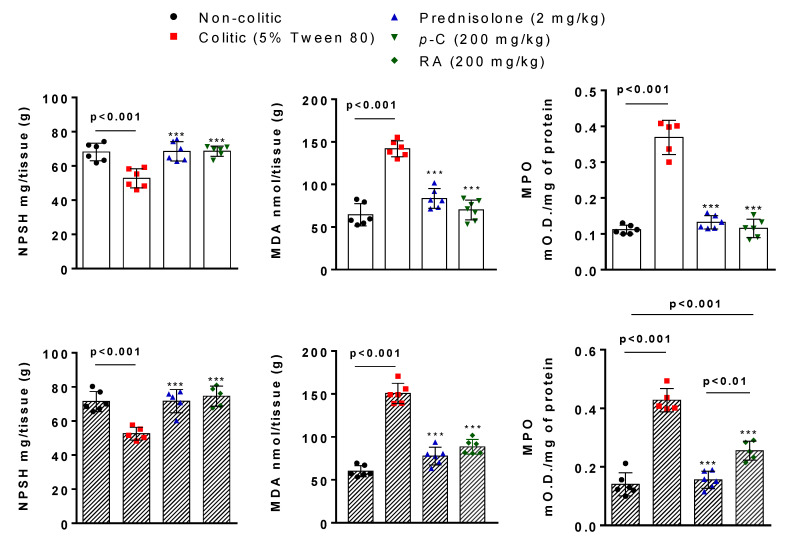
Effect of oral administration of *p*-C and RA on glutathione (GSH) (non-protein sulfhydryl groups (NPSH)), malondialdehyde (MDA), and myeloperoxidase (MPO) levels in colonic tissue from TNBS-induced ulcerative colitis in rats. Data are expressed as mean ± standard error, having been analyzed by one-way ANOVA followed by Dunnett’s or Tukey’s test. *** *p* < 0.001, compared to 5% Tween 80 or 0.9% NaCl control groups (*n* = 5–6).

Here, we evaluated the role of *p*-C and RA in the antioxidant system modulation during intestinal inflammation. Colonic tissues from the colitic group demonstrated a significant reduction (*p* < 0.001) of GSH levels (milligram of non-protein sulfhydryl groups (NPSH) per milligram of tissue) when compared to the non-colitic group. Treatment with *p*-C (200 mg/kg) or RA (200 mg/kg) restored (*p* < 0.001) GSH to baseline levels when compared to control groups (Figure 2).

**Figure 3:**
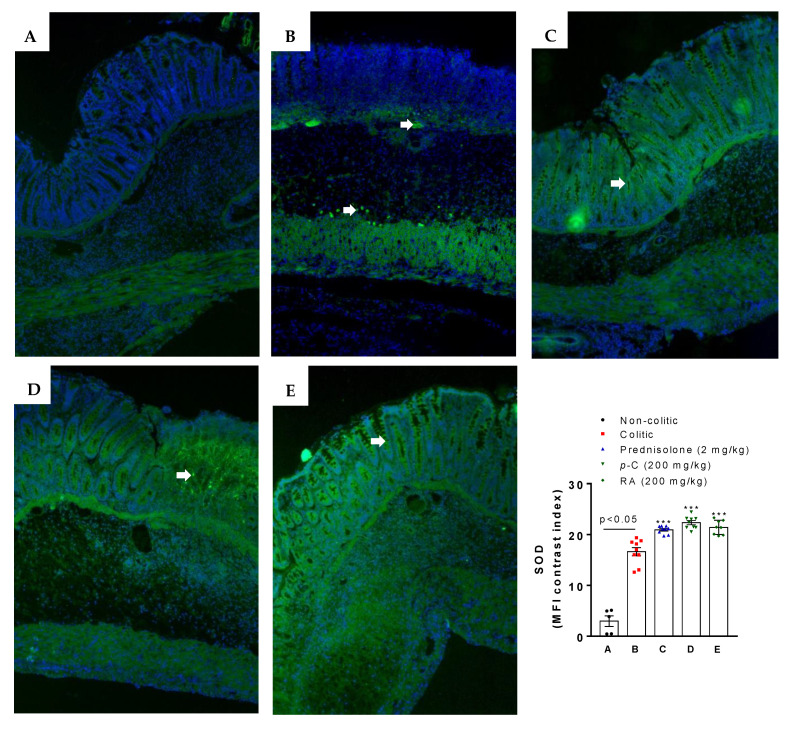
Representative photomicrographs of superoxide dismutase (SOD) with immunoreactivity marked in green (white arrows) and 4′,6-diamidino-2-phenylindole (DAPI) nuclear counterstained in blue from rats submitted to TNBS-induced ulcerative colitis. (**A**) Non-colitic group; (**B**) colitic group; (**C**) prednisolone (2 mg/kg); (**D**) *p*-C (200 mg/kg); (**E**) RA (200 mg/kg). Scale: 100 μm. Magnification: 400×. Data are expressed as mean ± standard error, having been analyzed by one-way ANOVA followed by Dunnett’s or Tukey’s test. *** *p* <0.001, compared to colitic control group (*n* = 5–9 fields/3 per group). MFI: median fluorescence intensity.

Immunofluorescence-assessed SOD labeling demonstrated an enhancement (*p* < 0.05) of fluorescence intensity in the intestinal tissue from the colitic group when compared to the non-colitic group. *p*-C orRA, on the other hand, intensified (*p* < 0.001) SOD fluorescence when compared to both colitic and non-colitic groups (Figure 3).

To support the interrelation between inflammation and oxidative stress, we assessed lipid peroxidation by MDA levels (nanomole MDA per milligram tissue) and neutrophil infiltration determined through dosing MPO activity (mean optical density per milligram of tissue). Results showed that colitic groups presented higher amounts (*p* < 0.001) of MDA and enhanced (*p* < 0.001) MPO activity when compared to the non-colitic group. *p*-C (200 mg/kg) or RA (200 mg/kg) significantly decreased (*p* < 0.001) the assessed parameters in comparison to colitic groups. However, only *p*-C brought the assessed markers to baseline levels. Data analysis also showed differences between RA and prednisolone-treated groups (*p* < 0.01 vs. prednisolone; *p* < 0.001 vs. non-colitic) (Figure 2).

### 2.3. p-C and RA Decreased Pro-Inflammatory (IL-1β and TNF-α) and Increased Anti-Inflammatory (IL-10) Cytokines in a TNBS-Induced Colitis Model

**Figure 4:**
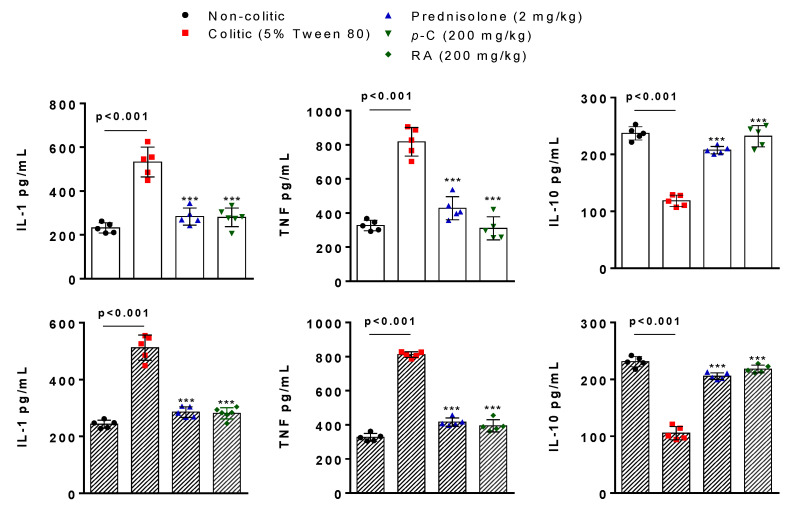
Effect of oral administration of *p*-C and RA on interleukin (IL)-1β, tumor necrosis factor (TNF)-α, and IL-10 levels in colonic tissue from TNBS-induced ulcerative colitis in rats. Data are expressed as mean ± standard error, having been analyzed by one-way ANOVA followed by Dunnett’s or Tukey’s test. *** *p* < 0.001, compared to 5% Tween 80 or 0.9% NaCl control groups (*n* = 5–6).

In this part of the study, we investigated a possible immunomodulatory effect on the basis of the levels of pro-inflammatory and anti-inflammatory cytokines. IL-1β and TNF-α levels (picogram of cytokine per milliliter) seemed to be enhanced (*p* < 0.001) in vehicle-treated groups (colitic groups) when compared to the non-colitic group. Treatment with *p*-C (200 mg/kg) or RA (200 mg/kg) decreased (*p* < 0.001) pro-inflammatory cytokine levels compared to the non-colitic group. When the anti-inflammatory cytokine IL-10 was evaluated, TNBS stimuli reduced (*p* < 0.001) its levels (picogram of cytokine per milliliter). However, *p*-C (200 mg/kg) and RA (200 mg/kg) increased (*p* < 0.001) IL-10 compared to colitic animals and restored it to baseline levels (Figure 4).

### 2.4. p-C and RA Maintained Constitutive Proteins of Intestinal Barrier (MUC-2 and ZO-1) in TNBS-Induced Colitis Model

**Figure 5:**
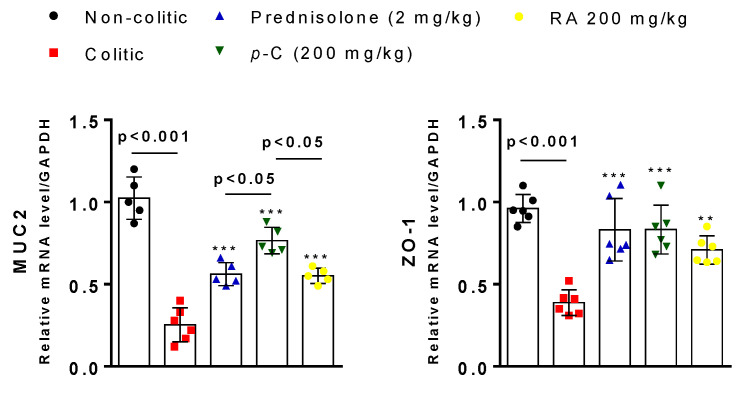
Effect of oral administration of *p*-C and RA in mucin type 2 (MUC-2) and zonula occludens 1 (ZO-1) expression in a TNBS-induced ulcerative colitis rat model. Data are expressed as mean of cell percentages ± standard error, having been analyzed by one-way ANOVA followed by Dunnett’s or Tukey’s test. *** *p* < 0.001, ** *p* < 0.01 compared to colitic control groups (*n* = 5–6).

To obtain additional information regarding the effects of *p*-C and RA on intestinal integrity, we carried out RT-qPCR and immunohistochemical assessments for two structural components related to the cytoprotection of intestinal barrier—the pre-epithelial (MUC-2) and epithelial (zonula occludens 1 (ZO-1)). Results showed that TNBS induced downregulation (*p* < 0.001) of MUC-2 and ZO-1 when compared to the healthy control groups, while treated groups increased (*p* < 0.001) their expression when compared to colitic groups. *p*-C brought MUC-2 to baseline expression (*p* > 0.05) when compared with the non-colitic group, and presented significant differences (*p* < 0.05) among the other groups (Figure 5).

**Figure 6:**
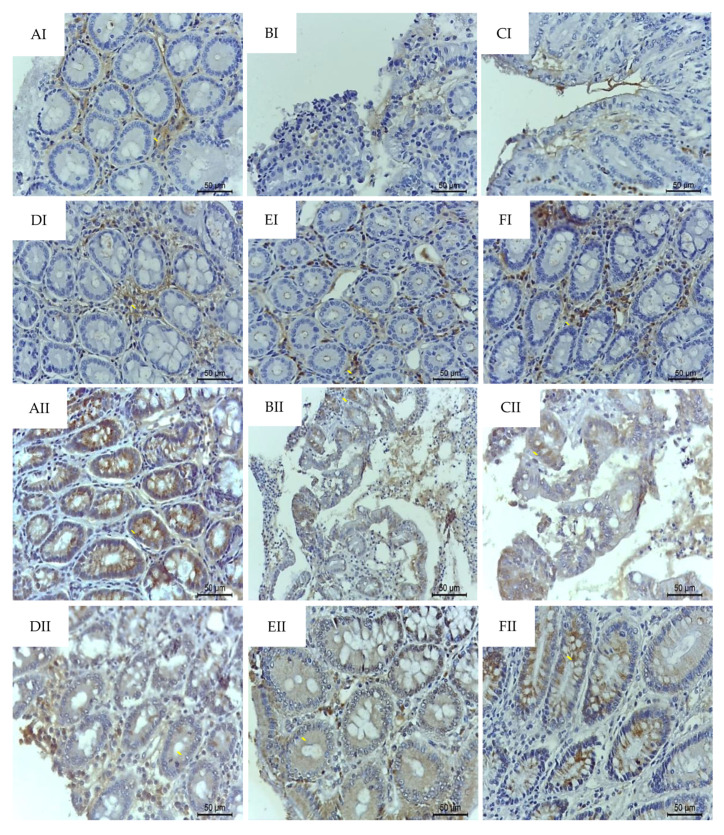
Photomicrographs with immunohistochemical staining (→) for MUC-2 (**I**) and ZO-1 (**II**) in colon samples from rats submitted to TNBS-induced ulcerative colitis. (**A**) Non-colitic; (**B**) colitic (5% Tween 80); (**C**) colitic (0.9% NaCl); (**D**) prednisolone (2 mg/kg); (**E**) *p*-C (200 mg/kg); (**F**) RA (200 mg/kg). Scale: 50 μm. 400×. MUC: mucin; ZO: zonula occludens.

Intense signs of MUC-2 depletion (*p* < 0.001) from mucin-producing cells and ZO-1 redistribution (*p* < 0.001) was observed by TNBS challenge in colitic animals when compared to healthy groups. On the other hand, oral treatment with *p*-C (200 mg/kg) or RA (200 mg/kg) reestablished (*p* < 0.001) their immunolabeling close to baseline levels, as shown in Table 2 and Figure 6.

### 2.5. p-C and RA Modulated T Cell Populations in Colon, Spleen, and Mesenteric on Lymph Nodes in a TNBS-Induced Colitis Model

**Figure 7:**
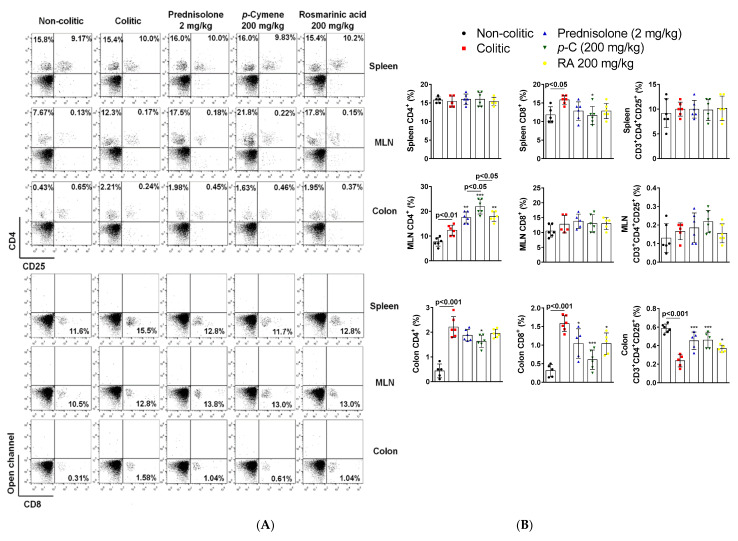
Effect of oral administration of *p*-C and RA in the modulation of T cell populations in a TNBS-induced ulcerative colitis rat model. (**A**) Representative dot plots from flow cytometry analysis (FACS) analysis of T lymphocytes gated for cluster of differentiation (CD)4^+^, CD8^+^, and CD3^+^CD4^+^CD25^+^ cells; (**B**) T cell percentages (%) in the assessed samples (colon, spleen, and mesenteric lymph nodes (MLN)). Data are expressed as mean ± standard error percentage of the total number of cells, having been analyzed by one-way ANOVA followed by Dunnett’s or Tukey’s test. *** *p* < 0.001, ** *p* < 0.01, * *p* < 0.05 compared to colitic control groups (*n* = 5–6).

Flow cytometry analysis (FACS) of T cell population in the spleen, mesenteric lymph nodes (MLN), and colon samples was also undertaken in this ulcerative colitis protocol. Results revealed no difference (*p* > 0.05) in the frequency of cluster of differentiation (CD)4^+^ and CD3 ^+^ CD4^+^CD25^+^ T cells across groups in the spleen. However, the colitic groups showed higher numbers (*p* < 0.05) of CD8^+^ T cells compared to the non-colitic group in this organ. Prednisolone (2 mg/kg) or RA (200 mg/kg) presented only a decreasing tendency (*p* > 0.05) in those levels, and only *p*-C (200 mg/kg) was able to significantly decrease (*p* < 0.05) CD8^+^ cells when compared to colitic groups. In MLN, results showed that CD4^+^ T cells were increased (*p* < 0.01) in the colitic groups when compared to the non-colitic group. Prednisolone, *p*-C, or RA led to an increase (*p* < 0.01, *p* < 0.001, and *p* < 0.01, respectively) of this T cell subtype when compared to colitic groups. Nevertheless, *p*-C seemed to be significantly different (*p* < 0.05) from all treated-groups. CD8^+^ and CD3 ^+^ CD4^+^CD25^+^ cell frequencies were not altered (*p* > 0.05) in the MLN. Finally, in colonic samples, CD4^+^ and CD8^+^ T cells were enhanced (*p* < 0.001) in colitic groups when compared to healthy controls. Prednisolone, *p*-C, and RA were able to decrease (*p* < 0.05, *p* < 0.001, *p* < 0.05, respectively) CD8^+^ cells, but only *p*-C decreased (*p* < 0.05) colon CD4^+^ cells in parallel to the colitic group. CD3 ^+^ CD4^+^CD25^+^ T cells were decreased (*p* < 0.001) in the colitic group in comparison to the non-colitic group, while treatment with prednisolone, *p*-C, or RA demonstrated enhanced (*p* < 0.001, *p* < 0.001, *p* < 0.05, respectively) frequency after comparison with the colitic group (Figure 7).

### 2.6. Effect of p-C and RA on the Gene Expression of COX-2, IFN-γ, iNOS, SOCS3, and NFκB-p65

**Figure 8:**
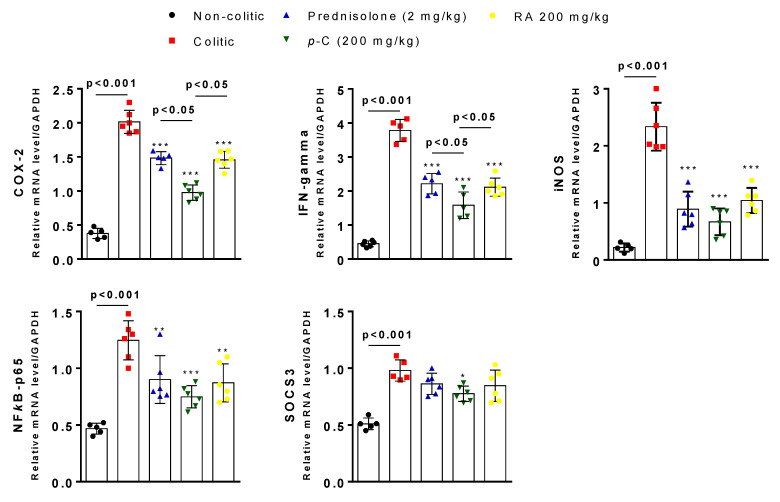
Effect of oral administration of *p*-C and RA in cyclooxigenase 2 (COX-2), interferon (IFN)-γ, inducible nitric oxide synthase (iNOS), suppressor of cytokine signaling 3 (SOCS3), and nuclear transcription factor kappa B subunit p65 (NFκB-p65) expression on TNBS-induced ulcerative colitis rat model. Data are expressed as mean of cell percentages ± standard error, having been analyzed by one-way ANOVA followed by Dunnett’s or Tukey’s test. *** *p* < 0.001, ** *p* < 0.01, * *p* < 0.05 compared to colitic control groups (*n* = 5–6).

Finally, we performed RT-qPCR analysis in order to assess gene expression of different inflammatory markers as well as proteins from the intestinal barrier. Colitic and non-colitic groups presented differences (*p* < 0.001) in the expression pattern of cyclooxigenase 2 (COX-2), IFN-γ, inducible nitric oxide synthase (iNOS), nuclear transcription factor kappa B subunit p65 (NFκB-p65), and suppressor of cytokine signaling 3 (SOCS3). Prednisolone, *p*-C, and RA negatively regulated COX-2 (*p* < 0.001), IFN-γ (*p* < 0.001), and NFκB-p65 (*p* < 0.01, *p* < 0.001, *p* < 0.01, respectively) expression, but only *p*-C decreased (*p* < 0.05) SOCS3 expression when compared to the colitic group. Furthermore, when comparing *p*-C with RA or even the prednisolone group, we found a statistically significant difference (*p* < 0.05) when COX-2 and IFN-γ gene expression was analyzed (Figure 8).

## 3. Discussion

Several of the animal models used to study IBDs involve genetic manipulations, infections, or chemical agents, e.g., induced by haptenization [36,37,38]. TNBS is a hapten that mimics inflammation induced by T cell response, in particular with a Th1 phenotype, resulting in IFN-γ release and macrophage activation [36]. This model shares significant properties with Crohn’s disease, as well as histopathological, biochemical, and clinical characteristics of intestinal lesions that emulate features of ulcerative colitis frequently observed in humans [38,39,40].

Using this model, we demonstrated that intestinal anti-inflammatory activity for both *p*-C and RA is due to a decrease in all assessed parameters. Similar effects were previously found for monoterpene 1,8-cineol and polyphenol eupatiline [27,41]. In addition, *Rosmarinus officinalis* L. extract, a major source of RA, has already shown to have an intestinal anti-inflammatory effect in the same model [42]. A similar occurrence has also been found with the *p*-C-containing species *Thymus vulgaris* L. (thyme) and *Origanum vulgare* L. (oregano) [43]. Histomorphological evaluation of tissues from rats subjected to *p*-C and RA treatment showed decreased lesion area and neutrophilic infiltrate. Neutrophils play an effective role in defense against microorganisms [44], however, their pathogenic role associated with IBDs has already been addressed [45,46]. Neutrophil activation promotes ROS accumulation and the release of MPO, an indirect marker of neutrophil infiltration [44,47]. One of the main effects of MPO is acting as a free radical and, due to the activation of matrix metalloproteinases, promoting fibrous layer weakening and disruption of intestinal epithelial tissue [48]. Both *p*-C and RA were able to decrease MPO activity. These results corroborate the findings in the histological assessment, and thus indicate that intestinal anti-inflammatory effect might be related to a decrease in the migration of inflammatory cells. The phenolic compound paeoniflorin and monoterpene geraniol have demonstrated protective effects on colonic tissue related to decreased MPO activity [49,50], similar to what was demonstrated by RA in a dextran-induced IBD model [51].

IBDs are constantly associated with overproduction of free radicals that contribute to the generation of oxidative stress [52]. Therefore, we investigated whether the intestinal anti-inflammatory effects of *p*-C and RA was related to antioxidant capacity. IBDs and their animal models are well known for reduced glutathione (GSH) levels and enhanced superoxide dismutase (SOD) activity in colon tissues [53,54]. Clinical and experimental evidence suggests that antioxidant impairment leads to increased MDA levels, a toxic product and the main marker of lipid peroxidation [55,56]. Our results showed that both substances tested here were able to decrease MDA and restore GSH to baseline levels, pointing to an antioxidant effect. When SOD immunolabeling was analyzed, data demonstrated enhanced immunofluorescence in the colitic group in parallel to the non-colitic group. These findings may suggest that *p*-C and RA surpass basal antioxidant mechanisms mediated by this enzyme after inflammatory challenge. Monoterpene 1,8-cineol in the acute phase of TNBS-induced intestinal inflammation demonstrated restoration of GSH levels under pre- and post-treatment conditions [24]. In addition, resveratrol, a polyphenol, had a colonic protective effect associated with antioxidant mechanisms that involved an increase in GSH and a decrease in MDA levels [57]. Furthermore, monoterpene geraniol also restored GSH levels and enhanced SOD activity in inflamed tissues [50].

Deregulation in production and inhibition of pro-inflammatory cytokines has also been implicated in the pathogenesis of IBDs [58]. NFκB is identified as one of the key regulators in this immunological setting through its ability to promote the expression of pro-inflammatory molecules (i.e., iNOS and COX-2) and cytokines (IL-6, IL-1β, TNF-α, etc.) [59]. IL-1β and TNF-α may alter epithelial permeability and facilitate the infiltration of inflammatory cells contributing to tissue damage. Furthermore, they mediate chronic intestinal inflammation by promoting the accumulation of IL-17A-secreting innate lymphoid cells and Th17 CD4^+^ cells [60,61,62]. Studies have revealed increased frequencies of activated cytotoxic T cells (CD8^+^) in active stages of IBDs close to the epithelial cells that may further exacerbate the inflammatory process through the production of TNF-α and IFN-γ, leading to the increased influx of luminal antigens through epithelial lesions [63,64]. TNF-α has been a potential target for the treatment of IBDs [65], with drugs acting as immunobiological agents proposed to counteract its actions [66]. In order to regulate intestinal inflammation, regulatory T cells (Treg, CD3^+^CD4^+^CD25^+^, or CD4^+^CD25^+^CD127^low^FoxP3^+^) take part in immune homeostasis and play a pivotal role in maintaining peripheral tolerance through the production of IL-10, IL-35, and TGF-β. Treg cell-mediated suppression is already known to be lower in patients with ulcerative colitis [67]. IL-10 is crucial to suppress cytokine release by activated macrophage and to block INF-γ production in Th1 cells [58]. During ulcerative colitis, IL-10 receptor polymorphisms have been associated with a lack of response to standard treatment [68]. Along with IL-10, SOCS3 is expressed by immune cells and may act as a regulator of NFκB-mediated cytokine release through the interference of the JAK/STAT pathway [69]. Increased SOCS3 expression has been detected in mouse models of IBD and in biopsies of ulcerative colitis patients where it seems to limit the extent of inflammation [70,71]. Both compounds tested in this study were able to decrease IL-1β and TNF-α, as well as bring IL-10 to basal levels in colon tissues. Thus, the findings found here point to a possible immunomodulatory effect, which may be attributed to a compensatory mechanism against colonic injury involving the modulation of T cells, represented by fewer amounts of CD8^+^ T cells in the spleen and colons that is positively correlated with a decreased expression of IFN-γ, while Treg cell frequency stayed close to control in the gut. It was also presented that *p*-C and RA showed an increase of CD4^+^ T cells in the mesenteric lymph nodes. The upregulation of CD4^+^ and B cells in the MLN may contribute to gut homeostasis and attenuation of colitis while interacting with Treg cells. CD4^+^ T cells might also lead to IL-10 production in the gut-associated lymphoid tissues to cause regulation of inflammatory process [72]. Furthermore, *p*-C and RA downregulated NFκB transcription and therein the regulation of their responsive genes, e.g., COX-2 and iNOS, that were evaluated in this study. Such effects possibly play a central role in reducing mucosal inflammation, preventing it from becoming uncontrolled in treated groups. *p*-C appears to be more effective in this context by inhibiting TNBS-induced inflammation and presenting a smaller frequency of colonic CD4^+^ cells and splenic CD8^+^ cells as well as SOCS3 downregulation in colon samples. D’Alessio et al. [73] studied the monoterpene d-limonene and demonstrated that its intestinal anti-inflammatory effect is related to a significant decrease in serum TNF-α concentrations via NFκB pathway inhibition. Studies with geraniol corroborated this study [50]. Moreover, treatment of colitic animals with *p*-C-rich medicinal species also decreased pro-inflammatory cytokines IL-1β, IL-6, GM-CSF, and TNFα mRNA levels [43]. Similar immunomodulatory effects have been shown for polyphenol paeoniflorin and flavonoid liquiritigenin [49,74].

Given the antioxidant and immunoregulatory effects induced by the compounds used here, we investigated a possible cytoprotective activity related to intestinal barrier integrity. The colon has a single layer of epithelial cells adjacent to the lumen where microbiota and luminal content reside. The contact of microorganisms with the epithelium is limited by a mucus biofilm, composed mainly by mucins (MUCs) produced by goblet cells [75,76]. MUCs consist of a major fragment containing a central protein and a large number of attached oligosaccharides. MUC-1, MUC-2, MUC-3, and MUC-4 might be found in colon section [77,78]. Evidence shows a decrease in mucus layer thickness during ulcerative colitis that is linked to changes in the genes responsible for MUC-2 expression, the most abundant mucin found in the intestine. These events are also accompanied by increased colonization of mucolytic bacteria and increased microbial penetration into lamina propria [79]. Moreover, MUC-2 knockout mice develop spontaneous colitis and are more susceptible to haptenization-induced intestinal inflammation [80,81]. Our results demonstrated an increase of MUC-2 in tissues of animals treated with the test substances and a restoration of their levels concerning the non-colitic group. These results might explain the attenuation of injury severity in treated groups, and point to intestinal cytoprotection due to mucus maintenance. The cranberry polyphenol-rich extract showed a similar effect against intestinal inflammation in association with probiotics of the genus *Akkermansia* spp., increasing MUC-2 mRNA in proximal colon samples [82]. Increased mucus secretion also provided a favorable environment for *Akkermansia* spp. to colonize intestinal mucosa and promote cytoprotection [83]. In addition, Taira et al. [84] also reported the role of dietary polyphenols in association with MUC-2 in rat stools submitted to the same experimental model.

Attached to the mucus layer, intestinal epithelium restricts the free passage of toxic molecules and microorganisms throughout the lumen, allowing selective paracellular passage through cell junctions [85]. Cell junctions are composed of multiple proteins, such as occludin, tricellulin, and claudins, and their intracellular portions interact with peripheral cytoplasmic membrane proteins, the so-called ZO-1, -2, and -3 [86]. ZO, in turn, interact with F-actin and myosin II, anchoring themselves to the cytoskeleton. Their association is highly dynamic and may play an important role in the regulation of the epithelial barrier [87]. Studies with epithelial cells from ZO-1-deficient mice pointed out that protein loss caused a delay in junction assembly and intestinal barrier dysfunction [88]. Furthermore, TNF-α and IFN-γ are known to inhibit occludin promoter gene [89] and lead to ZO-1 and claudin-1 redistribution [90,91]. An increase of ZO-1 marked cell and gene expression was seen for groups treated with *p*-C and RA. These findings may be correlated with the increased expression of MUC-2, which may lead to pre-epithelial protection and hinder inflammation development. In addition, a decrease in TNF-α levels and IFN-γ expression might be associated with a reduced cytokine-induced ZO-1 redistribution that protects the intestinal barrier from disruption. Studies with the polyphenols quercetin, gallate epigalocatequine, and resveratrol have been effective to prevent indomethacin-induced ZO-1 depletion [92]. In addition, polyphenol-rich propolis extract showed enhanced AMPK and ERK signaling, which led to increased expression of ZO-1 in human colon cells [93]. Until now, no data had been found in the literature that demonstrated MUC-2 and ZO-1 modulation promoted by terpenes in animal models or IBD patients.

## 4. Materials and Methods

### 4.1. Chemicals

*p*-Cymene (1-isopropyl-4-methylbenzene) and rosmarinic acid [(R) O-(3,4-dihydroxycinnamoyl)-3-(3,4-dihydroxyphenylactic acid)] were purchased from Sigma-Aldrich (Saint Louis, MO, USA) and solubilized in 5% Tween 80 and 0.9% NaCl, respectively. Substances used as standard drugs were solubilized according to the manufacturer’s specifications. Other chemicals included: 2-4-6-trinitrobenzene sulfonic acid (TNBS) (Sigma-Aldrich, Saint Louis, MO, USA), 1-methyl-2-phenylindole (Sigma-Aldrich, Saint Louis, MO, USA), 4′,6-diamidino-2-phenylindole (DAPI) (Sigma-Aldrich, Saint Louis, MO, USA), 5-5′-dithiobis-2-nitrobenzoic acid (DTNB) (Sigma-Aldrich, Saint Louis, MO, USA), ethylenediaminetetraacetic acid (EDTA) (Sigma-Aldrich, Saint Louis, MO, USA), hexadecyltrimethylammonium bromide (HTAB) (Sigma-Aldrich, Saint Louis, MO, USA), antibodies (Santa Cruz Biotechnology, Dallas, TX, USA), bovine serum albumin (BSA) (Sigma-Aldrich, San Louis, MO, USA), ELISA cytokine kits (R&D Systems, Minneapolis, MN, USA), ethanol (Merck, Darmstadt, Germany), prednisolone (Sigma-Aldrich, Saint Louis, MO, USA), hydrogen peroxide (Sigma-Aldrich, Saint Louis, MO, USA), ketamine (Vetanarcol, São Paulo, Brazil), o-dianisidine dihydrochloride (Sigma-Aldrich, Saint Louis, MO, USA), tween 80 (Merck, Darmstadt, Germany), Trisma buffer (Sigma-Aldrich, Saint Louis, MO, USA), potassium chloride (Sigma-Aldrich, Saint Louis, MO, USA) and xylazine (Dorcipec^®^, MSD saúde animal, São Paulo, Brazil).

### 4.2. Animals

All animal procedures followed the Animal Research: Reporting of In Vivo Experiments (ARRIVE) guidelines and the international principles for laboratory animal studies [94]. The Animal Use Ethics Committee of the Federal University of Paraíba also approved (15 Feb 2017) all experiments (CEUA UFPB 110/16). Wistar rats (*Ratus norvegicus*), 200–250 g, obtained from Prof. Dr. Thomas George Animal Facility (IPeFarM/UFPB) were used and kept at a controlled temperature (22 ± 1 °C; light/dark 12/12 h, water ad libitum). Rats were euthanized according to Resolution 1000/2012 of Federal Council of Veterinary Medicine with recommendations of the American Veterinary Medicine Association, preceded by general anesthesia with ketamine (150 mg/kg) and xylazine (20 mg/kg) intraperitoneally and after observation of the absence of the corneal reflex, supplemented with 1 mmol/kg potassium chloride, intravenously.

### 4.3. Trinitrobenzene Sulfonic Acid (TNBS)-Induced Intestinal Inflammation in Rats

The experimental protocol described by Morris et al. [95] was conducted with some modifications. After fasting for 24 h, rats were anesthetized with 2% xylazine hydrochloride and 5% ketamine hydrochloride for rectal administration of TNBS (10 mg per animal dissolved in 0.25 mL of 50% ethanol) with the aid of a 2 mm diameter probe, which was inserted around 8 cm inside the rectum. Following the administration of TNBS, animals were kept for 10 min upside down. Each group of animals was pre-treated orally with 5% Tween 80 and 0.9% NaCl (colitic groups), 2 mg/kg prednisolone (standard control group), and *p*-C or RA (25, 50, 100, and 200 mg/kg) 48, 24, and 1 h before TNBS administration and 24 h after inflammation induction. Then, after 48 h of TNBS administration, rats were anesthetized for blood collection and euthanized. Colonic segments were collected and photographed for ulcerative area (UA) quantification and macroscopic score determination. General parameters such as diarrhea and colon weight/length ratio were also evaluated. An untreated non-colitic group was added to the experiment whose animals were not submitted to inflammation induction. Intestinal injury score was assessed according to a scale previously described by Bell et al. [96]: (1) focal hyperemia, no ulcer; (2) ulceration, no hyperemia/bowel wall thickening; (3) ulceration, inflammation at one site; (4) ulceration, inflammation at 2 or more sites; (5) major injury > 1 cm; 6–10 major damage > 2 cm. The percentage of injury inhibition (%) was calculated as described:

(1) $$\%\;Lesion\;inhibition\left(\frac{Sample\;UA\;\times \;100}{Colitic\;group\;UL\;}\right)-100$$

To calculate the weight/length ratio parameter, the following formula was used:

(2) $$\frac{Colon\;weight\;\left(g\right)}{Colon\;lenght\;\left(\mathrm{cm}\right)}$$

Diarrheal status was determined from the Evacuation Index (EI) for 36 h until euthanasia. Stools were counted and classified as solids, semisolids, and liquids. The EI was calculated as follows:

(3) EI=Σ (nº of solid stools×1)+(nº of watery stools×2)+(nº of liquid stools×3)

### 4.4. Histological Analysis

Colon samples were fixed in ALFAC solution (37% formaldehyde; 80% alcohol; 37% acetic acid) for 24 h at room temperature, then dehydrated in an ethanol gradient and clarified with xylol. After processing, tissues were embedded in paraffin and sectioned (3 or 5 μm). Sections were stained with hematoxylin–eosin (HE) and optical microscopy was employed for a random and double-blind examination.

### 4.5. Determination of Reduced Glutathione (GSH) Levels

The method described by Faure and Lafond [97] was employed. Colon samples were homogenized with 5% trichloroacetic acid (1:20 *m*/*v*). To the homogenate, we added 320 µL of distilled water and then centrifuged the mixture (3000 rpm for 15 min at 4 °C). For GSH detection, we used 40 μL supernatant, 80 μL 0.4 M Tris buffer (pH 8.9), and 20 μL DTNB (5,5-dithiobisnitrobenzoic acid) for absorbance reading measured at 420 nm. Results were expressed by non-protein sulfhydryl groups (NPSH) units per gram (g) of tissue.

### 4.6. Determination of Malondialdehyde (MDA) Levels

MDA content was measured using Esterbauer and Cheeseman protocol [98]. Samples were homogenized in Trisma buffer (20 mM Tris-HCl, 1:5 *m*/*v*) and centrifuged at 10,000 rpm for 10 min at 4 °C. To the supernatant, we added 750 μL of 10.3 mM 1-methyl-2-phenylindole in acetonitrile and 225 μL 37% HCl. The samples were placed in a water bath at 45 °C for 40 min and centrifuged again. Supernatants were used to determine MDA levels measured at 586 nm. Results were expressed as MDA nanomole per gram (g) of tissue.

### 4.7. Determination of Myeloperoxidase (MPO) Activity

The assessment of MPO activity was conducted using the protocol described by Krawisz et al. [99]. Colonic segments were homogenized in hexadecyltrimethylammonium bromide (HTAB) buffer (0.5% in 50 mM sodium phosphate buffer (pH 6.0), 1:20 *m*/*v*), which acted as a detergent, lysing the neutrophil granules containing myeloperoxidase. The material was centrifuged at 4500 rpm for 12 min at 4 °C, and samples were subjected to a triple freezing and thawing process to facilitate enzyme release. Then, 50 μL of supernatant was collected, to which 150 μL of the reaction buffer (o-dianisidine hydrochloride, 50 mM phosphate buffer, and 33% H_2_O_2_) was added. The reading was then performed at 450 nm. Results were expressed as mean optical density (mOD) per milligram of tissue.

### 4.8. Determination of IL-1β, TNF-α, and IL-10 Levels

IL-1β, TNF-α, and IL-10 were determined in colon samples using commercial ELISA kits (R&D Systems, Minneapolis, MN, USA). After an 18 h coating with primary antibodies, we performed blocking with reagent diluent, and samples were added in duplicate and then incubated at 37 °C for 2 h. Biotinylated sheep polyclonal antibodies (anti-IL-lβ, anti-TNF-α, or anti-IL-10) were used and after 1 h (room temperature). Then, after washing, 100 µL of streptavidin–HRP-conjugated and solution A and B of the commercial ELISA kit were added for 20 min. The enzyme reaction was stopped with H_2_SO_4_ (1N) stop solution. Absorbance was measured at 450 nm, and results expressed as picogram of cytokine per milliliter.

### 4.9. Immunofluorescence

Sections of 3 μm (n = 5–9 fields per slide/3 per group) were prepared from paraffin-embedded colonic tissues. Samples were deparaffinized in xylene and washed in graded concentration series of ethanol. Sections were submitted to antigen retrieval using 10 mmol/L sodium citrate solution for 20 min at 95 °C. The autofluorescence background was reduced by incubating the sections with 0.1% Sudan black (diluted in 70% ethanol) for 20 min at room temperature. Sections were subjected to primary antibody incubation for superoxide dismutase (SOD; 1:200, Santa Cruz Biotechnology, Dallas, TX, USA) in 1% normal goat serum overnight at 4 °C, subsequently washed with phosphate-buffered saline (PBS) with 0.2% Triton X-100 (Sigma-Aldrich, Saint Louis, MO, USA), and incubated with Alexa Fluor 488-conjugated goat anti-rabbit secondary antibody (1:500 in 1% BSA). DAPI (4′,6-diamidino-2-phenylindole; Sigma-Aldrich, Saint Louis, MO, USA) was used to stain nuclear material, and then slices were mounted with Vectashield medium. Fluorescent images were obtained on a laser scanning microscope (model 710, Carl Zeiss, Oberkochen, Germany), and tissue reactivity was assessed by computerized densitometry of digital images. Average densitometric values were calculated and expressed as median fluorescence intensity (MFI) using ImageJ version 1.4 (NIH, Bethesda, MD, USA). Three specimens per group and five fields per slide were considered.

### 4.10. Immunohistochemical Analysis

Colon sections (3 μm; *n* = 5 fields per slide/3 per group) were obtained and transferred to silanized slides (Dako, Glostrup, Denmark), dewaxed, and hydrated. Slides were washed with 0.3% Triton X-100 in phosphate buffer, treated with 3% hydrogen peroxide, and incubated overnight at 4 °C with primary antibodies for mucin-type 2 (MUC-2, 1:200) and zonula occludens 1 (ZO-1, 1:200) (Santa Cruz Biotechnology, Interprise, São Paulo, Brazil). After washing, slides were incubated with secondary streptavidin–HRP-conjugated antibody (1:500, Biocare Medical, Concord, CA, USA) for 30 min, and immunoactivity for MUC-2 and ZO-1 was carried out (Trek Avidin-HRP label + Biocare Medical, CA, USA). Samples were visualized under an optical microscope (Leica DM750, Schweiz, Switzerland) with Qwin system coupled to a camera (Leica ICC50 HD, Wetzlar, Germany). The intensity of immunostaining was determined for each animal using 5 random fields (real area 327.68 × 245.76 μm). Quantification was performed by AVSoft Bioview version 4.0.1.

### 4.11. Flow Cytometry Analysis

Spleens were collected and tissue dissociation was performed using a lysing buffer (0.15 M NH_4_Cl; 0.1 mM EDTA; 12 mM Na_2_HCO_3_) and 2 frosted glass slides for frictioning. Mesenteric lymph node (MLN) cell suspensions were prepared by mechanical dissociation using a stainless steel screen. Colon samples were washed in RPMI-1640 (Thermo Fisher, Waltham, MA, USA), and incubated in calcium- and magnesium-free Hank’s balanced salt solution with 5 mM EDTA, dithiothreitol (0.29 mg/mL), penicillin (1000 U/mL), and streptomycin (0.1 mg/mL) for 90 min at 37 °C with continuous stirring. After centrifugation, resuspension in RPMI-1640 containing 25 mM HEPES, penicillin (1000 U/mL), streptomycin (0.1 mg/mL), and deoxyribonuclease II (0.1 mg/mL) (Sigma-Aldrich, Saint Louis, MO, USA) was performed. Cell isolation from all samples were carried out after washing in PBS and filtering through a 70 μm nylon filter. After that, resulting cells were incubated for 30 min with BD Horizon Fixable Viability Stain (San Diego, CA, USA) and a mix of monoclonal antibodies: fluorescein isothiocyanate (FITC) CD3 (rat, 0.5 mg/mL, BD Pharmingen, cat. 555274, San Diego, CA, USA), allophycocyanin (APC) CD4 (rat, 0.2 mg/mL, BD Pharmingen, cat. 553051, San Diego, CA, USA), phycoerythrin (PE) CD8a (rat, 0.2 mg/mL, BD Pharmingen, cat. 553033, San Diego, CA, USA), and PE-Cy7 CD25 (rat, 0.2 mg/mL, BD Pharmingen, cat. 553866, San Diego, CA, USA). For each sample, 30,000 events were acquired using a fluorescence cell counter (BD FACSCanto II, Piscataway, NJ, USA). Lymphocytes were distinguished by their different forward-scatter (FSC) versus side-scatter (SSC) profiles and were gated and scored. Then, CD4^+^, CD8^+^, and CD3^+^CD4^+^CD25^+^ populations were analyzed using FlowJo v9.7.6 software (Treestar, Ashland, OR, USA).

### 4.12. Gene Expression by RT-PCR

Colon samples were homogenized and total RNA was isolated using Trizol (Invitrogen, Carlsbad, CA, USA). After milling, RNA extraction was carried out using a SV Total RNA isolation system kit (Promega Corporation, Madison, WI, USA), according to the manufacturer’s specifications. Quantification of RNA samples was performed using Thermo Scientific NanoDrop (Waltham, MA, USA). From total RNA, we performed single-strand complementary deoxyribonucleic acid (cDNA)-reverse transcription reaction synthesis using a High-Capacity cDNA Reverse Transcription Kit (Applied Biosystems, Foster City, CA, USA) and a Step One Plus thermocycler (Applied Biosystems, Foster City, CA, USA) using fluorophore SYBR Green PCR Master Mix (Applied Biosystems, Foster City, CA, USA). The following gene-specific primers sequences (Fw: forward/Rv: reverse, Table 3) were used:

GAPDH gene was used as a reference gene and results were expressed as fold change according to the value calculated from delta-delta Ct (^ΔΔ^Ct) method for a gene expressed in experimental versus control conditions (reference/constitutive).

### 4.13. Statistical Analysis

Parametric data were expressed as mean ± standard deviation (SD)/error (SE) or non-parametric data as median (minimum/maximum values). Data were subjected to variance analysis (one-way ANOVA) followed by Dunnett’s or Tukey’s test (parametric), or Kruskal–Wallis test followed by Dunn’s test (non-parametric). *p* < 0.05 was considered statistically significant, and GraphPad Prism Software 7.0 was used for data processing.

## 5. Conclusions

Our data demonstrated that *p*-C and RA present intestinal anti-inflammatory activity related to antioxidant and immunomodulatory effects, which are followed by cytoprotective mechanisms, acting on either the pre-epithelial or epithelial barrier. These outcomes suggest a potential use of these isolated compounds to the treatment of intestinal inflammation.

## Figures and Tables

**Table 1:** Effect of oral administration of *p*-C and RA in the acute phase of trinitrobenzene sulfonic acid (TNBS)-induced intestinal inflammation. Data are expressed as mean ± SD or median (minimum/maximum values).

Groups	Dose (mg/kg)	Ulcerative Area (mm^2^)	% Lesion Inhibition	Lesion Score	Weight/Length (mg/cm)	Evacuation Index (EI)
Non-colitic	-	ND	-	ND	101.5 ± 16.04	370
Colitis (Tween)	-	318.6 ± 38.41 ^###^	0	7.5 (6–9) ^###^	191.2 ± 33.77 ^###^	751 ^###^
Prednisolone	2	91.5 ± 42.19 ***	70.0	3 (2–5) ***	131.4 ± 18.44 ***	412 ***
*p*-Cymene	25	255.3 ± 44.53 **^,abcd^	19.7	5 (3–6) *	150.9 ± 16.22 *	578 ***^,ad^
	50	144.0 ± 43.91 ***^,d^	54.7	4.5 (3–5) **	137.9 ± 31.33 **	509 ***^,ad^
	100	111.2 ± 15.74 ***^,d^	65.0	4 (2–5) **	130.0 ± 24.41 **	450 ***^,d^
	200	58.53 ± 14.16 ***	81.6	2.5 (1–4) ***	119.3 ± 17.03 ***	388 ***
						
Non-colitic	-	ND	ND	ND	117.1 ± 24.78	381
Colitis (NaCl)	-	341.1 ± 42.99 ^###^	0	8 (7–10) ^###^	182.5 ± 12.29 ^###^	773 ^###^
Prednisolone	2	114.3 ± 17.04 ***	66.6	3 (2–6) ***	122.3 ± 11.38 ***	420 ***
Rosmarinic acid	25	262.5 ± 23.92 **^,acd^	19.1	6 (3–7) *	155.0 ± 30.04 *	613 ***^,a.d^
	50	184.7 ± 12.21 ***^,ad^	44.1	5.5 (4–8) *	146.6 ± 21.36 **	553 ***^,ad^
	100	155.1 ± 39.02 ***	54.2	5 (3–7) **	144.7 ± 11.14 **	488 ***
	200	112.0 ± 15.26 ***	67.2	4 (2–6) ***	133.4 ± 9.89 ***	470 ***

For parametric data, we used one-way ANOVA followed by Dunnett’s and Tukey’s post-tests. For non-parametric data, we used Kruskal–Wallis test and Dunn’s test. * *p* < 0.05, ** *p* < 0.01, *** *p* < 0.001 compared to colitic groups; ^###^ *p* < 0.001 compared to non-colitic group; ^a^ *p* < 0.05 compared to prednisolone group; ^b^ *p* < 0.05 compared to 50 mg/kg group; ^c^ *p* < 0.05 compared to 100 mg/kg group; ^d^ *p* < 0.05 compared to 200 mg/kg group (*n* = 7–10). ND = not detectable.

**Table 2:** Effect of oral administration of *p*-C and RA on MUC-2 and ZO-1 immunostaining in colonic tissue from TNBS-induced ulcerative colitis rat model.

Treatments	Dose	MUC-2 (μm^2^)	ZO-1 (μm^2^)
Non-colitic	-	2130 (332.1–2536.3)	725 (136.4–1020.3)
5% Tween 80	10 mL/kg	745 (222.3–993.0) ^###^	305 (90.5–489.1) ^###^
0.9% NaCl	10 mL/kg	690 (221.4–823.4) ^###^	295 (120.4–401.0) ^###^
Prednisolone	2 mg/kg	1700 (789.0–1944.1) ***	675 (156.5–903.3) ***
*p*-Cymene	200 mg/kg	1903 (866.0–2531.5) ***	685 (488.1–789.3) ***
Rosmarinic acid	200 mg/kg	1675 (659.3–1722.1) ***	550 (222.4–697.5) ***

Data are expressed as median (minimum/maximum values), having been analyzed by Kruskal–Wallis test and Dunn’s test. *** *p* < 0.001 compared to colitic group; ^###^ *p* < 0.001 compared to non-colitic group (*n* = 5 fields/3 per group). MUC: mucin; ZO: zonula occludens.

**Table 3:** List of assessed genes and primer sequences evaluated by RT-qPCR in colon samples from TNBS-induced ulcerative colitis protocol.

Gene	Primer Sequences
MUC-2 ZO-1	Fw: GATAGGTGGCAGACAGGAGA Rv: GCTGACGAGTGGTTGGTGATTG Fw: GGGGCCTACACTGATCAAGA Rv: TGGAGATGAGGCTTCTGCTT
COX-2	Fw: CGCTTCTCCCTGAAACCTTAC Rv: GTAGAGGGCTTTCAACTCTGCA
IFN-γ iNOS	Fw: ATGAGTGCTACACGCCGCGTCTTGG Rv: GAGTTCATTGACAGCTTTGTGCTGG Fw: GCTACACTTCCAACGCAACA Rv: GTGGGAGGGGTAGTGAT
SOCS3	Fw: CCTCCAGCATCTTTGTCGGAAGAC Rv: TACTGGTCCAGGAACTCCCGAATG
NFκB (p65)	Fw: CTGGCAGCTCTTCTCAAAGC Rv: CCAGGTCATAGAGAGGCTCAA
GAPDH	Fw: CCATCACCATCTTCCAGGAG Rv: CCTGCTTCACCACCTTCTTG

## Abbreviations

APC	Allophycocyanin
BSA	Bovine serum albumin
CD25	Cluster of differentiation 25
CD3	Cluster of differentiation 3
CD4	Cluster of differentiation 4
CD8	Cluster of differentiation 8
cDNA	Complementary deoxyribonucleic acid
COX-2	Cyclooxygenase-2;
DTNB	5-5’-dithiobis-2-nitrobenzoic acid
EDTA	Ethylenediaminetetraacetic acid
EI	Evacuation index
ERK	Extracellular signal-regulated kinases
FITC	Fluorescein isothiocyanate
GSH	Reduced glutathione
H2O2	Hydrogen peroxide
HE	Hematoxylin-eosin
HTAB	Hexadecyltrimethylammonium bromide
IFN-γ	Interferon-gamma
IL-10	Interleukin 10
IL-1β	Interleukin 1 beta
iNOS	Inducible nitric oxide synthase
MDA	Malondialdehyde
MPO	Myeloperoxidase
MUC2	Mucin type 2
NFκB-p65	Factor nuclear kappa B subunit p65
NPSH	Non-protein sulfhydryl
PBS	Phosphate-buffered saline
PE	Phycoerythrin
RNA	Ribonucleic acid
SOCS3	Suppressor of cytokine signaling 3
SOD	Superoxide dismutase
TNBS	2,4,6-trinitrobenzene sulfonic acid
TNF-α	Tumoral necrosis factor alpha
UA	Ulcerative area
ZO-1	Zonula occludens
